# Evaluation of the Outcomes of Coronectomy Procedure versus Surgical Extraction of Lower Third Molars Which Have a High Risk for Inferior Alveolar Nerve Injury: A Systematic Review

**DOI:** 10.1155/2021/9161606

**Published:** 2021-11-12

**Authors:** Nedal Abu-Mostafa, Lulwah M. AlRejaie, Fahad A. Almutairi, Ruba A. Alajaji, Maram M. Alkodair, Nourah A. Alzahem

**Affiliations:** ^1^Oral and Maxillofacial Surgery and Diagnostic Science Department, Riyadh Elm University, Riyadh, Saudi Arabia; ^2^Riyadh Elm University, Riyadh, Saudi Arabia; ^3^Majmaah University, Al Majma'ah, Saudi Arabia; ^4^King Saud University, Riyadh, Saudi Arabia; ^5^King Khalid University, Abha, Saudi Arabia

## Abstract

**Results:**

No study reported permanent inferior alveolar nerve injury (p-IANI) regarding coronectomy; however, transient inferior alveolar nerve injury (t-IANI) was reported in 0–2.20% of successful coronectomy and 0–8% of failed coronectomy. Postextraction t-IANI ranged from 0% to 16.66% while p-IANI from 0% to 3.63%. In 5 studies, root migration occurred in 2% to 85.3% of cases and the distance rate was 2.33–3.43 mm at 6 months postoperatively; then the migration gradually decreased and stopped at 12 months.

**Conclusion:**

This systematic review revealed that coronectomy is an efficient alternative for the management of impacted 3rd M with a high risk of IANI. Patients who got antibiotics postcoronectomy procedures had lower infection rates than those who did not receive antibiotic therapy. We recommend further research on coronectomy with longer follow-up periods to assess the retained roots' long-term outcomes and to assess the effect of antibiotics administration on postcoronectomy infection rate. This systematic review is registered under number CRD42020198394.

## 1. Introduction

Mandibular third molars are the most commonly impacted teeth and the leading cause of various pathologies, from mild infection and inflammation to severe cystic lesions requiring surgical removal of the teeth [1]. Extraction of the impacted third molar is among the most frequently performed surgical procedures, with prevalence ranging from 35.9% to 58.7% [2]. As mandibular third molar roots are near the inferior alveolar canal, they have a high probability of causing neurosensory disturbances if the inferior alveolar nerve gets traumatized due to any treatment of the impacted tooth [1, 3]. Third molars close to the inferior alveolar canal must be extracted carefully. Many clinicians may avoid extraction of these mandibular third molars to avert injury to the inferior alveolar nerve (IAN) [4]. The incidence of neurapraxia following mandibular third molar extraction is about 1% to 5%, and the rate of persistent IAN involvement has been reported in up to 0.9% cases. However, more than 30% of the inferior alveolar nerve injuries (IANI) have been reported in confirmed cases of high-risk IAN involvement [5]. Apart from IAN's involvement, several cases of damage to the lingual nerve during surgical removal of mandibular third molars have been reported [6]. Coronectomy procedure or deliberate vital root retention is a solution to avoid nerve injury. This technique is used to remove the crown portion of the third molar, keeping the roots intact and thus posing no harm or damage to the inferior alveolar nerve [7].

To select the best possible procedure, it is required to perform a complete clinical and radiographic assessment. Panoramic radiograph can show the proximity of the mandibular molar roots to the inferior alveolar nerve. Rood and Shehab [8] summarized the radiographic indicators of the close relationship between the lower third molar and the inferior alveolar canal. The first group of indicators on the third molar includes root darkening, root deflection, root narrowing, and bifid apex. Other signs present on the inferior alveolar canal include interruption of the white lines, diversion, and narrowing of the canal.

Cone-Beam Computed Tomography (CBCT) provides a better analysis of the association between dental roots and the mandibular canal than does panoramic radiograph [9]. However, a meta-analysis by de Toledo Telles-Araújo et al. [10] has not found strong evidence that the radiographic assessment by CBCT reduces neurosensory disturbance after lower third molar removal compared to panoramic radiograph. The overuse of CBCT is not recommended and should be reserved for third molars with a high risk of trauma to the inferior alveolar nerve. The radiation dose from CBCT is significantly higher compared to conventional dental radiography techniques [11].

For third molars with a high risk of inferior alveolar nerve injury, coronectomy can protect the nerve, unlike surgical extraction; however, the risk of postoperative infections was similar [12]. Coronectomy which also is called “intentional partial odontectomy” [13] has been given special attention in the past few decades, as many successful outcomes of this procedure have been reported [14].

This study aims to systematically review clinical studies that evaluated the effectiveness and the complications of coronectomy procedure as an alternative for surgical extraction of impacted third molars that have a high risk of trauma to the inferior alveolar canal.

## 2. Materials and Methods

A systematic review of the published studies was conducted to evaluate the clinical outcomes of the coronectomy procedure compared to the surgical extraction of impacted third molars. This study was registered in the University Research Center, and the registration number was SRS/2020/13. The Institutional Review Board of the University approved the study, and the approval number was SRS/2020/13/195/184.

### 2.1. Search Strategy

This review included only the studies that were published in English, and the full texts were available. Electronic databases of PubMed (https://pubmed.ncbi.nlm.nih.gov), SCOPUS (https://www.scopus.com), and ScienceDirect (https://www.sciencedirect.com) were used to search for articles. The last access was on June 5, 2020. Searching databases included medical subject heading (MeSH) terms (such as Impacted, third molars, coronectomy, odontectomy, inferior alveolar nerve injury). Additionally, a search string was used: [(coronectomy AND “impacted third molars”) OR (Extraction AND “impacted third molars”) OR (coronectomy AND “impacted third molars”) OR (odentectomy AND “inferior alveolar nerve injury”) OR (Extraction AND “inferior alveolar nerve injury”) OR (odentectomy AND “inferior alveolar nerve injury”)].

### 2.2. Focused Question

Does the coronectomy procedure have better outcomes compared to the surgical extraction of impacted third molars close to the inferior alveolar canal, which can lead to a severe risk of nerve injury? This focus question will be addressed using the PICO approach: P (population): Patients at any age who had an impacted 3rd molar in close proximity to the IAN based on radiographic signs on panoramic radiograph or CBCT images. I: (intervention): coronectomy and surgical extraction. C (comparison): the comparison of coronectomy with surgical extraction of these third molars. O (outcome): primary outcome: inferior alveolar injury (IANI). Secondary outcomes: root migration, infection, and failure.

### 2.3. Inclusion and Exclusion Criteria

The review included studies that evaluated the effects of coronectomy and compared its outcomes with complete surgical extraction of impacted third molars next to the inferior alveolar canal that would pose a high risk of nerve injury. We included randomized clinical trials (RCTs), controlled clinical trials (CCTs) and prospective cohort studies (PCSs), prospective (PS), and retrospective (RS) studies with a control group. These studies had a minimum of 10 coronectomy procedures performed with a follow-up period of at least two months. Case reports, in vitro studies, comments to authors, literature reviews, and prospective or retrospective studies without a control group were excluded.

### 2.4. Data Extraction and Analysis

Two reviewers performed data extraction and analysis. The reporting followed the PRISMA statement, and the checklist was filled out for every evaluated study. The following parameters were collected from the selected articles and analyzed: authors, publication year, number of patients, mean age, gender, study design, number of surgical extractions and coronectomy procedures, abnormal sensitivity of the inferior alveolar nerve, abnormal sensitivity of lingual nerve, pain onset, swelling, the occurrence of infection or alveolar osteitis, roots migration following coronectomy, the need for reoperation to remove the roots, and the follow-up period. Cochrane Collaboration's tool was used to identify the risk of different forms of bias, including selection, performance, detection, attrition, and reporting bias (Table 1). The performance of meta-analysis of the results was impossible due to the heterogeneity of the studies' outcomes.

## 3. Results

**Figure 1:**
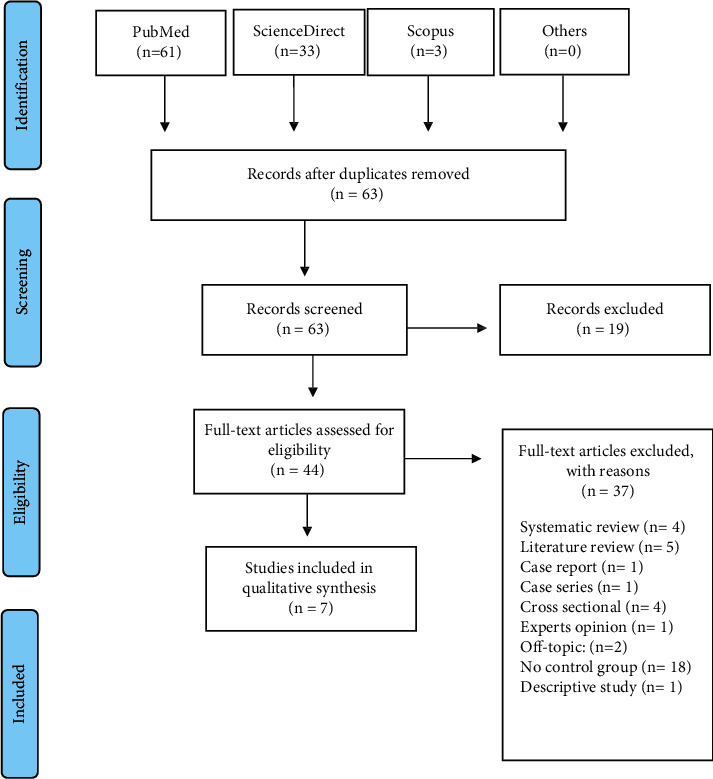
PRISMA flowchart.

A total of 97 articles relevant to this topic were retrieved from 3 different databases. Duplicates were excluded, resulting in 63 papers. Nineteen articles were excluded because they were irrelevant to subject of the review. Accordingly, 44 full-text articles were assessed for eligibility, and 37 were excluded (Figure 1). Only 7 studies fulfilled the inclusion criteria for the quality synthesis: 3 RCTs [16–18] and 4 CCTs [5, 15, 19, 20] (Table 2).

### 3.1. Studies Characteristics

The sample size of teeth undergoing coronectomy ranged from a minimum of 15 teeth [18] to a maximum of 102 teeth [5]. The included mean age was between 24.9 years [18] and 32.4 years [5]. All studies considered a control group. Six studies monitored both sexes [5, 16–20]. The secondary outcomes including numbers and percentages of patients, teeth, incidence of pain, infection, alveolar osteitis, failure, and other variables are available in Table 3.

### 3.2. Surgical Interventions and Medical Treatments

The coronectomy procedures were performed on a minimum of 15 teeth [18] to a maximum of 171 teeth [17]. In the control group, extraction procedures were done on samples of teeth ranging from a minimum of 15 [18] to a maximum of 178 [17]. The indications and the provided postoperative medical therapy were specified in most of the included studies. Antibiotics were prescribed in three studies postoperatively [15, 18, 19] and preoperative chlorhexidine mouthwashes were used in two studies [15, 16]. Analgesics were prescribed in two studies [15, 17], and discutient was mentioned in one study postoperatively [19]. Researchers in one study did not prescribe medications [20], and one study did not mention any pharmacological treatment [5].

### 3.3. Coronectomy Failure

The failure criteria were defined and specified in five studies [5, 15–18]. Four studies reported failed coronectomy defined as loosening, mobility, or dislodgement of the roots during or after the decrowning procedure [15, 16, 17, 19]. One study stated failed coronectomy if the remaining roots needed to be extracted due to infection occurrence [5]. Regardless of the criteria used to assess the failure, in studies reporting the failure, the least percentage of coronectomy failure was 0% in the study of [18] and the highest percentage was 38.3% in the study of [16]. For certain cases, root dislodgements during the treatment led to a change in plan from coronectomy to surgical removal. Furthermore, they noticed that women with conically rooted teeth that narrowed within the nerve canal was a factor predicting failure of coronectomy.

### 3.4. Clinical Outcomes

Transient inferior alveolar nerve injury (t-IANI) in successful coronectomy ranged from 0% [15, 16, 18, 19] to a maximum of 2.20% [20] (Table 2). On the other hand, the percentages of t-IANI in failed coronectomy were 8% and 6.25% in the studies of Renton et al. [16] and Leung and Cheung [17] respectively, respectively. No study has reported permanent inferior alveolar nerve injury (p-IANI) regarding coronectomy.

In the study done by Hatano et al. [5], one out of 102 patients in the coronectomy group had t-IANI (0.98%). In the control group (*n* = 118), 6 patients (5.08%) had IANI, and 3 of them were diagnosed with p-IANI. Leung and Cheung [17] found IANI in one patient (0.6%) in the coronectomy group (*n* = 155). However, in the study of Cilasun et al. [15], no patients in the coronectomy group (*n* = 88) had IANI, while 2 out of 87 patients (2.29%) in the control group had t-IANI. In the study of Renton et al. [16], 19 IANI (18.6%) were found in the control group (*n* = 102). Kang et al. [19] reported 6 patients (10.91%) with IANI in the control group (*n* = 55), with 4 of them being t-IANI and 2 being p-IANI. No IANI was observed in the study group (*n* = 55). The study carried out by Yan et al. [20] found lingual nerve injury (LNI) in 1 patient (2.04%) in the control group (*n* = 47) and 0% in the coronectomy group. No other LNI was found in the coronectomy and extraction groups of the other studies [5, 15, 16, 18]. One study did not mention LNI in their clinical outcomes [19].

Root migration was investigated in five studies [5, 16–19]. Three studies [17–19] reported the distance of root migration in millimeters by different displacement times, which varied across studies. The means of retained roots movement over 3 months were 1.9 mm [17], 2.19 mm [19], and 2.97 [18]. Over 6 months, the means of root movement were 2.33 mm [17], 2.91 mm [19], and 3.43 mm [18]. Over 12 months, the means of root movement ranged from 2.97 mm [17] to 3.15 mm [19]. Two studies did not mention root migration in their clinical outcomes [15, 20]. The main follow-up period ranged from a minimum of 6 months [18, 20] to a maximum of 36 months [19].

The pain was a clinical finding in 7 studies [5, 15–20]. Pain percentage ranged from a minimum of 1.1% [15] to a maximum of 41.9% [17]. The study of Kang et al. [19] found that postoperative pain resolved more rapidly in the coronectomy group compared to the extraction group (*p* < 0.001). A study conducted by Singh et al. [18] comparing pain intensity between the coronectomy group and the odontectomy group did not show statistically significant group differences, and the *p* values were found to be 0.024 preoperatively, 0.353 on the first day, and 0.243 on the 7th day. The study done by Renton et al. [16] recorded pain in 22 patients (21.6%) in the control group (*n* = 102), 8 patients (13.8%) in the coronectomy group, and 4 patients (11.1%) in the failed coronectomy group, with no statistically significant differences between the three groups. Leung and Cheung [17] reported pain in 57.3% (102/178) of the teeth in the control group and 41.9% (65/155) in the coronectomy group in the 1st week postoperatively. The difference was statistically significant (*p*=0.005). Nevertheless, the difference between the two groups at 1 to 24 months after surgery was nonsignificant. On the other hand, Hatano et al. [5] found that the percentage of postoperative pain (18.6%) (19/102) was higher in the coronectomy group compared to extraction group (6.78%, 8/118), and the difference was significant (*p*=0.012). In the study by Cilasun et al. [15], only one case experienced pain (1.1%) in the coronectomy group (*n* = 88), whereas no cases reported pain in the control group.

The infection was investigated in the 7 studies, with the percentage ranging from a minimum of 0.98% [5] to a maximum of 10.99% [20] in coronectomy groups, and from 0.98% [16] to 10.2% [20] in the extraction group. Yan et al. [20] found the infection rate in coronectomy group (10.9%) was close to the control group (10.2%), while Renton et al. [16] found higher infection rate in the coronectomy group (3/58) (5.2%) than the control group (1/102) (0.98%). Conversely, Hatano et al. [5] recorded less infection rate the coronectomy group (1/102) (0.98%) than the control group (4/118) (3.39%). In Leung and Cheung's study [17], infection rate was 5.8% (9/155) in the coronectomy group and 6.7% in the control group (12/178). Cilasun et al. [15] found that the rate of infection was 1.1% in the coronectomy group (1/88) and 0% in the control group (87). Singh et al. [18] and Kang et al. [19] observed no incidence of postoperative infection in the coronectomy and control groups.

Hatano et al. [5] recorded 8.47% of Alveolar Osteitis (AO) in the extraction group and 1.96% in the coronectomy group, and the difference was significant (*p*=0.039). In the same way, Leung and Cheung [17] found no cases of AO in the coronectomy group, whereas in the control group, 2.8 percent (5/178) of cases had AO in the first postoperative week. This was a statistically significant difference (*p*=0.036). However, in the studies of Renton et al. [16], Cilasun et al. [15], and Kang et al. [19], no significant differences were found between the two groups regarding AO.

## 4. Discussion

Coronectomy has been introduced as a new clinical procedure to minimize the risk of IANI upon removal of lower third molars. Despite that, due to the lack of evidence-based clinical trials, effectiveness and longstanding outcomes are yet to be verified by further research [21]. This systematic review was designed to evaluate the effectiveness and the complications of coronectomy procedure as an alternative to surgical extraction of impacted third molars, which carries a high risk of trauma to the inferior alveolar canal. The surgical technique described previously by Pogrel et al. [7] was also used by Singh et al. [18] with a high success rate. CBCT, as a three-dimensional imaging technique, can show a stable and accurate anatomical relationship between the inferior alveolar canal and the roots of third molars with a high risk of nerve injury. Therefore, it can indicate whether coronectomy should be selected to treat impacted third molars to protect the inferior alveolar nerve [22].

In studies that were included in this systematic review, the overall rate of postcoronectomy failure was 10% or less, although high failure rates of 16.36% in the study of Kang et al. [19] and 38.8% in the study of Renton et al. [16] were also reported.

The percentage of t-IANI following the coronectomy procedure was found to be 8.3% or less [16]. This finding is inconsistent with a recent study done by Pitros et al. [23], who reported 4.3% of t-IANI following the coronectomy and 18.6% following extractions.

Five studies reported t-IANI after extractions which ranged from 2.29% [15] to 16.66% [16]. This systematic review revealed that the percentages of t-IANI were less in coronectomy procedures than extractions. None of the studies in this review recorded p-IANI following coronectomy. However, 4 studies reported p-IANI following extractions [5, 16, 17, 19] and ranged from 1.68% to 3.63%.

In Mukherjee et al. [24], LNI following coronectomy procedure was observed in one out of 20 patients (5%). In this review, LNI was not reported after coronectomy procedures in all the included studies. Thus, the incidence of lingual nerve injury was considered to be infrequent. On the other hand, LNI was reported in one case of extraction (2.04%) in the study of [20]. The average postcoronectomy pain was 19%, and the highest pain percentage (41.9%) was found in a study by Leung and Cheung in 2009. In a recent study done by Shokouhi et al. [25], the pain percentage reached 56.4%.

The infection rates following coronectomy procedures for patients who did not receive any antibiotic therapy in the studies by Renton et al. [16], Hatano et al. [5], Leung and Cheung [17], and Yan et al. [20] were 5.2%, 1.1%, 5.8%, and 10.99%, respectively. We found these rates higher than Cilasun et al. [15], Singh et al. [18], and Kang et al. [19] who administered antibiotics postoperatively, as their infection rates were 1.13%, 0%, and 0%, respectively. Pogrel et al. [7] described the need to apply antibiotics in the pulp chamber while performing the coronectomy procedure to reduce the incidence of postoperative complications.

Most studies have emphasized that coronectomy should be performed on vital teeth free of inflammation because pulpitis is likely to result in further apical disease [19]. Periapical infection was not reported with the retained roots in the included seven studies in this review.

Root migration or late eruption was a common consequence of coronectomy. According to Singh et al. [18] and Kang et al. [19], more than half of the roots migrated at a high rate during the first 3–6 months postoperatively, and subsequently the rate steadily dropped and ended migrating around 12 to 24 months due to bone deposition and connective tissue coverage. References [18, 19] observed the root migration and specified that the distance of root migration from the inferior alveolar canal ranged from 2.33 mm to 3.43 mm at the first postoperative six months of follow-up.

During three years of follow-up, Kang et al. [19] discovered that root migration distances in the female patients were greater than males. Moreover, they compared the effect of root morphology on migration distance following coronectomy procedure. The columnar roots moved faster than the enlarged roots in the 3rd and 6th postoperative months and the differences were significant (*p*=0.008 and *p*=0.045). They explained this finding by the greater bone resistance to the enlarged roots than conical roots during migration. In the same way, roots with incomplete apex migrate more quickly than completely formed roots. Furthermore, the roots of vertical and distoangular impacted third molars were more likely to move till being exposed to the oral cavity.

A recent study by Yan et al. [26] investigated root migration after coronectomy and the mean recorded migration distance was 4.05 ± 1.98 mm. They discovered that preoperative conditions of the lower third molar, such as impaction depth, retromolar space, and angulation, influence root migration from apex to crown. However, the patient's age was the most important factor in determining the total distance of root movement as younger patients have more chance for root migration. The same study monitored the migrated roots rotation in three dimensions and came up with some interesting results. The mean of roots rotation was recorded to be 13.24 ± 7.21°. They concluded that the number of roots and gender of the patients had no significant effect on the root complex's rotation. However, the preoperative angulation of the third molar was the main factor that influenced root rotation. The smaller angulation was linked to more distal rotation while larger angulation resulted in more mesial rotation following surgery. No root eruption was observed though many of roots had moved to the alveolar crest. Yan et al. [26] pointed out that the length of the root complex must be less than 7.6 mm, and the gap between the root and the alveolar crest must be at least ≥5 mm to avoid root exposure and the need for secondary surgery.

Lee et al. [27] used two-dimensional (2D) analysis by plain X-ray films and three-dimensional analysis (3D) by CBCT to investigate root migration after a coronectomy procedures. At 6 months after coronectomy, they discovered that 64% of the roots (21 of 33 instances) had migrated more than 2 mm in 2D analysis. However, in the 3D analysis at the same time interval, the mean migration distance was 4.11 mm. They studied the factors affecting migration and concluded that impacting the mandibular third molars horizontally rather than vertically would increase the likelihood of migration. The horizontal and mesial impactions provide a large space into which the root might move following coronectomy, whereas vertical and distal angulation has a little space. Furthermore, they realized that root migration increases when the impaction depth is superior rather than inferior, the root form is convergent rather than divergent, and the crown cutting is completed rather than incompletely because the root can still be physically prevented from moving during eruption if the cutting is done incorrectly.

Regarding the follow-up period, the mean duration across the included studies in our review was sufficient to assess IANI, LNI, pain, infection, and pulpal health; however, it was insufficient to assess root migration. Coronectomy has been shown to be safe for at least the first two years [17, 20]. However, this period is not enough to assess the late eruption that might occur up to 10 years after coronectomy. A more extended follow-up period is required to assess the outcomes of the retained roots that might erupt, cause a late infection, or need removal [16].

The percentage of the required reoperation due to root migration was low, ranging from 0% [16] to 20% [18]. The included studies suggested the need for further research with larger sample sizes and longer follow-up periods to determine the retained roots' long-term outcomes. Besides, some of the included studies did not describe the procedure steps done minutely, which is an essential factor that might influence the procedure's success. Thus, future studies should provide technical details about the procedure done.

## 5. Conclusion

The reviewed studies confirmed that coronectomy is an efficient alternative for managing impacted third molars with a high risk of IANI, as the coronectomy procedure has fewer complications compared to surgical extraction of those teeth. However, root migration requires an extended follow-up period. The maximum rate of root migration occurs within the first 6 months of coronectomy procedure and become gradually stable after 1 year. Moreover, infection rates after coronectomy were lower in the studies where patients were given antibiotics than in the studies where antibiotics were not given. As a result, we recommend more research be done on the influence of pre- or post-operative antibiotics on infection rates after coronectomy.

## Figures and Tables

**Table 1:** Risk of bias assessment with the recommended approach of Cochrane Collaboration.

Domain	Renton et al. [16] RCT	Hatano et al. [5] CCT	Leung and Cheung [17] RCT	Cilasun et al. [15] CCT	Singh et al. [18] RCT	Kang et al. [19] CCT	Yan et al. [20] CCT
Random sequence generation	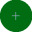	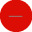	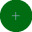	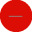	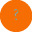	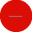	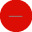
Allocation concealment	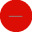	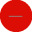	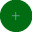	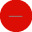	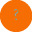	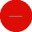	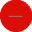
Blinding of participants and personnel	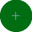	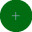	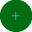	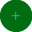	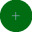	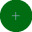	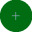
Blinding of outcome assessment	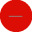	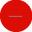	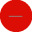	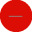	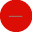	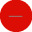	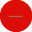
Incomplete outcome data	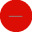	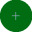	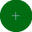	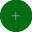	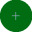	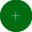	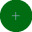
Selective reporting	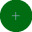	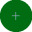	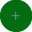	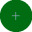	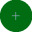	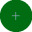	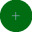
Other bias	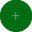	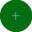	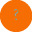	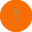	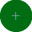	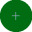	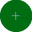

**Table 2:** Summarized data of the nerve injuries in the 7 included studies.

Authors and year	Study design	Success or failure of coronectomy	IANI in extractions	IANI in successful coronectomy	IANI in failed coronectomy	LNI
Renton et al., 2005	RCT	S: 58 (61.7%) F: 36 (38.3%)	19 (18.6%)	0%	t-IANI: 3 (8%) (mean 3 weeks)	0%
		E: NA	t-IANI: 17 (16.66%)			0%
			p-IANI (˃ 6 months): 2 (1.96%)			

Hatano et al., 2009	CCT	*S* = 97 (95%)	6 (5.08%)	t-IANI: 1 (0.98%)	0%	0%
		F = 5 (4.9%)	t-IANI: 3 (2.54%)			
			p-IANI: 3 (2.54%)			

Leung and Cheung, 2009	RCT	S: 155 (90.6%)	9 (5.1%) t-IANI: 6 (3.37%)	t-IANI: 1 (0.6%)	t-IANI: 1 (6.25%; 1/16)	0%
		F: 16 (9.4%)	p-IANI 3 (1.68%)			

Cilasun et al., 2011	CCT	*S* = 86 (97.7%)	t-IANI: 2 (2.29%)	0%	0%	0%
		*F* = 2 (2.3%)				

Singh et al., 2018	RCT	*S* = 15 (100%)	0%	0%	0%	0%
		*F* = 0				

Kang et al., 2019	CCT	S: 46 (83.63%)	6(10.91%) t-IANI: 4 (7.27%)	0%	0%	NA
		F: 9 (16.36%)	p-IANI: 2 (3.63%)			

Yan et al., 2020	CCT	S: 91 (97.84%) F: 2 (2.16%)	NA	t-IANI: 2 (2.20%)	NA	C: 0%
						E: 1 (2.04%)

RCT: randomized clinical trial; CCT: controlled clinical trial; C: coronectomy; E: extraction; S: success; F: failure; t-IANI: transient inferior alveolar nerve injury; p-IANI: permanent inferior alveolar nerve injury; LNI: lingual nerve injury.

**Table 3:** Summarized data of the other findings of the 7 included studies.

Authors and year	Gender	Age	Teeth no C or E	Pharmacological treatment: antibiotics, analgesics, CHX, others	Pain	Infection	Alveolar osteitis (AO)	Root migration rate	Follow-up and reoperation	Implication and conclusion
Renton et al., 2005	C:	C: 29.0 ± 6.47	C:94	Preoperative chlorhexidine mouth washes.	C:8 (13.8%)	C:3 (5.2%)	C: 12.1% (7/58)	(5/94) 5.3%	25 ± 13 months	Low risk of complications than extraction
	M 30; F 64	Failed C: 27.93 ± 5.8					Failed C: 11.1% (4/36)	<2 mm	Reoperation; 0	
	E:	E: 27.54 ± 5.5	E:102		E:22 (21.5%)	E:1 (0.98%)	E: 9.6% (10/102)			
	M 35; F 67						TX: irrigation with chlorhexidine and dressing with alveogyl (butyl aminobenzoate, eugenol, and iodoform)			

Hatano et al., 2009	C:	C: 32.36 ± 10.39	C:102	NA	C:	C: 1 (0.98%)	C: 1.96% (2/102)	(85.29%)	Follow-up; 13 months	Low risk of complications than extraction
	M 27; F 75				19 (18.6%) VAS Day of surgery: 59.6 Postoperatively Day 1: 37 Day 3: 29.8 Day 5: 20.2 Day 7: 7.6				Reoperation; 5 patients (4.90%)	
	E:	E: 32.19 ± 8.47	E:118		E:	E: 4 (3.39%)	E: 8.47% (10/118)			
	M 34; F 84				8 (6.78%) VAS Day of surgery: 47.7 Postoperatively Day 1: 32.6 Day 3: 18.7 Day 5: 17.8 Day 7: 2.8		TX: NA			

Leung and Cheung, 2009	M 70; F 101	C: 27.2 ± 7.3	C:171	No antibiotics were prescribed.	C: 65 (41.9%)	C:9 (5.8%)	E: 2.8% (5/178) of cases first postoperative week	3 months; 1.90 ± 1.23 mm	C: 10.6 ± 7.7 months	Low risk of complications than extraction.
					VAS: end of the 1st post-op week: 3.1 (SD, 1.9)		C: NA	12 months; 2.97 ± 1.47 mm	Failed C 11.4 ± 7.9 months	
		E: 26.2 ± 6.3	E:178	Analgesics: paracetamol and codeine for 3 days.	E: 102	E: 12 (6.7%)	TX: NA	24 months; 3.06 ± 1.67 mm	E: 7.7 ± 6.6 months	
					VAS: End of 1st post-op week: 3.7 (SD, 1.8)				Reoperation; 2 (1.17%) in month 9	

Cilasun et al., 2011	C: NA E: NA	C: 27.36 E: 27.19	C:88 E:87	Postoperative antibiotics: (amoxicillin clavulanate 625 mg, 2 × 1) and oral rinses (benzydamine HCl plus chlorhexidine gluconate, 2 × 1) for 5 days.	C: 1 (1.1%) E: 0%	C: 1 (1.1%) E: 0%	E (1/87) C: NO TX: irrigation and dressing with alveogyl (Septodont, France)	NA	C: 16.97 ± 12 months E: 17.62 ± 12 months Reoperation; 1 (1.13%) patient who underwent reoperation at her own decision	Effective alternative to extraction when there is a high risk of IAN injury

Singh et al., 2018	M 5; F 10	24.9 ± 3.933	C:15 E:15	Post-op antibiotics: capsule ampicillin 250 mg and capsule cloxacillin 250 mg and tablet metronidazole 400 mg 3 times daily Analgesics: ibuprofen 400 mg and paracetamol 325 mg 3 times daily for 3 days.	Pain intensity: C: VAS Pre-op: 19.10 1st day: 16.97 7th day: 17.33 E: VAS Pre-op: 11.90 1st day: 14.03 7th day: 13.67	C: 0% E: 0%	NA	1 day; 1.53 mm 1 month; 2.07 mm 3 months; 2.97 mm 6 months; 3.43 mm	Follow-up; 6 months Re-operation; 3 (20%) patients	Effective alternative to extraction

Kang et al., 2019	M 49 F 43	C: 26.5 E: 25.3	C: 55 E: 55	Postoperative antibiotics: (cephradine and metronidazole) and discutient prescribed for 3 days.	C (days): 2.61 ± 1.95	No infection	E: 5.45% (2/55)	3 months; 2.19 ± 0.80 mm	Follow-up; 36 months	Coronectomy should be considered superior to extraction in managing the risk of IANI.
					E (days): 3.40 ± 1.55		C: 1.82% (1/55)	6 months; 2.91 ± 0.87 mm	Reoperation; 5 (9.09%) patients	
							TX: wound debridement and irrigation.	12 months; 3.15 ± 0.90 mm		
								36 months; 3.19 ± 0.92 mm		

Yan et al., 2020	M 49; F 91 teeth (pts:121)	C: 27.20 ± 4.31	C: 93 E: 47	No	C VAS (mean): 1.99% ± 1.38 E: VAS (mean):1.84% ± 1.86	C: 10 (10.99%) E: 5 (10.2)	No significant difference between the two groups. TX: NA	NA	Follow-up; 6 months, Only 1 case (1.07%) of more than 6 months	Coronectomy had less influence on IAN function than conventional extraction.	

M: male; F: female (in gender and age columns); C: coronectomy; E: extraction; S: success; F: failure (in success and failure column); and NA: not available.

## Data Availability

This review included only the studies that were published in English, and the full texts were available. Electronic databases of PubMed (https://pubmed.ncbi.nlm.nih.gov), SCOPUS (https://www.scopus.com), and ScienceDirect (https://www.sciencedirect.com) were used to search for articles.
